# Oral Electricity and Vital Currents

**Published:** 1896-04

**Authors:** S. B. Palmer

**Affiliations:** Syracuse, N. Y.


### ORAL ELECTRICITY AND VITAL CURRENTS.

BY S. B. PALMER, M.D.S., SYRACUSE, N. Y.

THE January issue of the *Dental Cosmos* for 1896 and the *Dental Practitioner and Advertiser* for the same month contain a paper by the writer entitled "Dental Science Then and Now." This paper was written in answer to a challenge by the editor of the *Dental Practitioner*, as the following quotations from his editorial will show: "In this instance, we did desire to challenge an answer on the part of Dr. Palmer, and we are very glad that the attempt was successful, and that he was roused to the effort which the sound of the bugle provoked. Nothing but good can come of it, provided no feeling of personality enters into the discussion." Knowing, as we do, that Dr. Barrett does not allow discussion to break the bonds of friendship, we have only to look forward to the promised good. The epitome is a fair review of the paper, and an acknowledgment that it has made a considerable impress upon his mind. "But there are other points on which we can but doubt." Dr. Barrett has entered into this discussion for the benefit of the profession, and no doubt voices the sentiment of many other representative teachers and writers; therefore we use the points at variance as texts from which to elucidate our understanding. To save quotations we refer the reader to the paper above mentioned, which will give the substance in detail.

The paper contains answers to some of Dr. Black's conclusions, published in the *Dental Cosmos*, May, 1895, under the head of "The Physical Characteristics of the Teeth." In the discussion we classified teeth, defining them as physical and vital. By physical we mean any and all that had been extracted, those with devitalized pulps, including those fully matured, and all that have done receiving support from the pulp. Second or vital, immature, or teeth of children, and highly organized or sensitive teeth at any age. The first division have been so ably defined by Dr. Black that we need only to refer the reader to his article. The second class, we claim, are vital organs of the body, which receive nourishment and support from the pulp, which acts as an organ to mature a tooth, as other organs do to nourish and support other parts of the system. This view of the matter accounts for the systemic changes in the teeth due to ill-health, long sea-voyages, change of climate, effects of drugs, etc.

It includes constitutional differences not discernible under analysis or visible by the microscope. Let it be understood that we are now discussing matters upon the vital plane in the animal kingdom. Because we do not know just what life is we should not despair of searching for the laws which govern those conditions. This has been the study of the writer for years, and we now lay before the reader a few practical suggestions as the outcome of investigation.

This brings us to a full understanding of the hinderances in the way of receiving benefits from our teachings. Our opponents are still searching for light of the living among the dead. I quote the following: "Demonstrated facts cannot be successfully refuted by mere generalizations. They must be met by counter-facts as incontestably demonstrated by equally conclusive experiments. Physical proofs cannot be overthrown by abstruse metaphysical reasoning, and the world is waiting for some one to present a series of established tables that shall confute those of Dr. Black. He has made assertions, and apparently proved them, that are at variance with all our preconceived ideas. If he is right, we must look elsewhere than in the degree of calcification for the liability of teeth to decay."

The above shows plainly that the table and figures are based upon the physical plane. In all the discussions upon this subject investigators have failed to recognize the continuity of loss from elements to animals. We read in the history of creation, "And the Lord God formed man of the dust of the ground, and breathed into his nostrils the breath of life: and man became a living soul." This brief statement doubtless includes vastly more time than many suppose. Chemistry verifies the record. Where the simple elements of matter *are* there is the breath of life (chemical affinity) also. It touches atoms, and chemical compounds appear. Another breath,—lichens, grass, flowers, fruits, and trees abound. Again, this organized matter receives vivifying force from above, and man has had breathed into him the breath of life, and in him is incarnated all the uplifting forces and vital evolutions that preceded him.

And at this point I desire the reader to understand the cause of opposition. First, there is no recognized authority for the doctrines we set forth. Second, their adoption would call for a complete revision of text-books on physiology. Medicine would be more deeply interested than dentistry. As before mentioned, all matter upon the animal plane has been organized mainly by the agency of vegetable life, in which vital electrical currents performed a part, by which is meant the to-and-fro electrolytic currents which transmit the influence of solar heat and light from foliage to roots and return. This conductor is the sap, which holds in solution the elements required to build up and nourish every portion of the vegetable. The same principle appears in the higher animals. The blood contains the elements, and the various organs perform the office of distribution. In all immature teeth we see the principle mentioned in the growth of trees. The tubes in dentine are filled with the fluid containing the elements necessary to mature the organ. This fluid is in connection with the pulp, which furnishes the supply. It is not intended by nature that this current should be exposed to the action of external agents any more than that the brain should be affected by fractures of the skull. Those who oppose the electro-vital theory contend that gold does not constitute an element of a battery in a tooth unless it is adjacent to other metallic fillings. This would be true from a physical stand-point, when we consider that vital organic matter makes up the other elements of the battery, also that a negative plate of metal bears the same relations to nature's vital current that minerals do when taken into the stomach as food. The effect of metal upon immature dentine is to stay the process of calcification by thermal changes, which in organized currents combine thermal changes. Heat or warmth is life, the other extreme is death. Thus it is that dentine containing a large proportion of organic matter becomes a conductor of currents or temperature; the natural current being reversed, the dentine in contact with fillings is not matured, and caries is the effect.

With this understanding, we can see that by closing the dentinal tubes with some insoluble lining, thereby protecting the fluid in the dentine, the process of calcification will continue. We rarely find dentine sufficiently dense to resist external currents before the age of fourteen or fifteen years. As we approach the age when the teeth are matured the tables and figures given by Dr. Black apply as claimed.

The object of this writing is to draw a distinct line between teeth that are receiving support from the pulp and those deprived of such nourishment, either by devitalization or having received the required proportion of lime-salts to render dentine a non-conductor; in either case vitality has little to do in changing the physical condition of the dentine. We do not intend to controvert opposition. If the world is not yet ready to receive the doctrines herein set forth, we must await further investigation. The request that a like series of established tables shall be presented that shall confute those of Dr. Black. This is most unreasonable and unscientific, to insist that vital organic compounds shall be tried by physical laws. Who would believe that a like investigation of the skeletons of a robin and an English sparrow would or could determine which bird would endure the lowest temperature? Yet the world knows from observation. And so does the dental world know that teeth of children need filling-materials best adapted to their conditions. The laws of life no more fit matter upon the physical plane than Saul's splendid armor fitted David for the battle in which he was about to engage. All understand that mind influences matter. What is the connection? A locomotive stands upon the track, fired up and ready to start. The engineer has a mind to make it go. The order emanating from the brain is transmitted down to the throttle-valve through the various planes to come in touch with inert matter that matter had to pass through in evolution to come in touch with mind. Mind despatches an organized current to the muscles of the arm, the valve is opened, the engine obeys. Involuntary action of organs act upon the body, in respiration, heart action, assimilation, etc., according to the conditions, producing normal or abnormal effects. In this manner the dental organs conform to systemic influences.

The question of etiology of dental caries cannot be settled upon the physical plane. Carefully-conducted experiments on that line furnish tables, figures, and conclusions which teach that, "with our present knowledge, the only basis for selection and adaptation of filling-materials to classes of cases is the individual operator's judgment as to which he can so manipulate as to make most perfect filling, considering circumstances, his own skill, and durability of materials." Opposite we have the recorded testimony of clinical teaching that gold alone is not the best material (even in the most skilful hand) to use in filling the teeth of young patients, and practice seems to recognize this as a fact; and here the dental world seems to be waiting for tables and figures, which cannot be produced upon the animal plane. Let us return to the foot of the scale, take the simple elments, and with the evolution run to the top, and return, observing the principles of the first law and its conformity to each stage of the evolutions of matter. First, we have matter and chemical affinity which unites elements, and chemical compounds are the result. Second, minerals are organized, vegetation springs up, and in connection and direction organized currents take the place of the electrical current on the mineral plane. Third, organic matter combines, and animal life appears, with the organic current, now vitalized, preserving its involuntary functions, which in the vegetable kingdom brought forth flowers, fruits, trees, each after its kind, in animal bodies through the organs perform the same office, and still higher the vital current comes in touch with the soul and the Creator. Thus man thinks, wills, and through his organization acts as truly in conformity to the organized current as the telegraph-sounder acts under the the physical current. With this key to knowledge, many of the perplexed questions regarding physiology would appear simple.

